# Tumor necrosis as a poor prognostic predictor on postoperative survival of patients with solitary small hepatocellular carcinoma

**DOI:** 10.1186/s12885-020-07097-5

**Published:** 2020-06-29

**Authors:** Yi-hong Ling, Jie-wei Chen, Shi-hong Wen, Chao-yun Huang, Peng Li, Liang-he Lu, Jie Mei, Shao-hua Li, Wei Wei, Mu-yan Cai, Rong-ping Guo

**Affiliations:** 1 State Key Laboratory of Oncology in South China, Collaborative Innovation Center for Cancer Medicine, Sun Yat-sen University Cancer Center, Guangzhou, China; 2 Department of Pathology, Sun Yat-sen University Cancer Center, No. 651, Dongfeng Road East, Guangzhou, 510060 China; 3 Department of Anesthesiology, The First Affiliated Hospital, Sun Yat-sen University, Guangzhou, China; 4 Department of Hepatobiliary Oncology, Sun Yat-sen University Cancer Center, No. 651, Dongfeng Road East, Guangzhou, 510060 China

**Keywords:** Tumor necrosis, Prognosis, Small hepatocellular carcinoma

## Abstract

**Background:**

Small hepatocellular carcinoma (sHCC) is a special subtype of HCC with the maximum tumor diameter ≤ 3 cm and excellent long-term outcomes. Surgical resection or radiofrequency ablation provides the greatest chance for cure; however, many patients still undergo tumor recurrence after primary treatment. To date, there is no clinical applicable method to assess biological aggressiveness in solitary sHCC.

**Methods:**

In the current study, we retrospectively evaluated tumor necrosis of 335 patients with solitary sHCC treated with hepatectomy between December 1998 and 2010 from Sun Yat-sen University Cancer Center.

**Results:**

The presence of tumor necrosis was observed in 157 of 335 (46.9%) sHCC patients. Further correlation analysis showed that tumor necrosis was significantly correlated with tumor size and vascular invasion (*P* = 0.026, 0.003, respectively). The presence of tumor necrosis was associated closely with poorer cancer-specific overall survival (OS) and recurrence-free survival (RFS) as evidenced by univariate (*P* <  0.001; hazard ratio, 2.821; 95% CI, 1.643–4.842) and multivariate analysis (*P* = 0.005; hazard ratio, 2.208; 95% CI, 1.272–3.833). Notably, the combined model by tumor necrosis, vascular invasion and tumor size can significantly stratify the risk for RFS and OS and improve the ability to discriminate sHCC patients’ outcomes (*P* <  0.0001 for both).

**Conclusions:**

Our results provide evidence that tumor necrosis has the potential to be a parameter for cancer aggressiveness in solitary sHCC. The combined prognostic model may be a useful tool to identify solitary sHCC patients with worse outcomes.

## Background

As the second leading cause of cancer mortality worldwide, hepatocellular carcinoma (HCC) is an aggressive malignancy with its global incidence especially in Southeast Asia and Africa, with an increasing incidence in Europe and America [1–3]. Because of the persistent prevalence of hepatitis B virus (HBV) infection, HBV-related liver cirrhosis and/or HCC has become a main disease burden in China [4]. Currently, early detection and surveillance allow for the discovery of more small HCCs (sHCC, ≤ 3 cm in diameter) [5, 6].

Solitary sHCC is a special type of HCC with favorable long-term outcomes [7, 8].. Albeit surgical resection provides potentially curative therapies, disease recurrence is still frequently found in many patients postoperatively and less is known about the factors correlated with aggressive biological phenotype of sHCC [5–8]. It may be ideal to identify patients at high risk of tumor recurrence and/or poorer outcome, and to target close follow-up or postoperative adjuvant therapies in these sub-populations [9, 10]. Currently, the known clinicopathological factors for sHCC enable the identification and screening the patients at high risk, however, the reliable factors remain ill-defined [11, 12].

Traditionally, pTNM stage and histological grading systems are recognized as the most useful prognostic factors of sHCC. In addition, other features such as tumor size and vascular invasion are also utilized in clinical setting and found to be prognostic assessment of sHCC patients. Tumor necrosis is a common pathological feature of solid tumors, which is documented to be correlated with chronic ischemic injury due to the rapid growth of tumor [13–15]. The extent of tumor necrosis reflects the level of intra-tumor hypoxia. What’s more, increased cellular hypoxia is linked to the increased metastatic potential and worse outcome in solid tumors, as well as resistance to radio-chemotherapy [13, 16]. To date, the clinical and prognostic implication of tumor necrosis in solitary sHCC remain elusive [17, 18]. In the present study, we proposed to assess the prognostic value of tumor necrosis in solitary sHCC following hepatectomy and to demonstrate whether tumor necrosis can be regarded as a parameter for sHCC aggressiveness. In addition, we further aimed to construct a clinicopathologic model with risk factors to predict the prognosis of sHCC patients.

## Methods

### Case selection

The data of the pathologically proven and non-distant-metastasis solitary sHCC patients (335 cases) in Sun Yat-sen University Cancer Center (Guangzhou, China) were retrospectively collected between December 1998 and 2009. In the current study, the selected patients were undergoing surgical resection (not ablation or transplantation) as the first therapy course. The eligibility criteria including: (1) solitary sHCC with the diameter ≤ 3 cm; (2) hepatitis B surface antigen positive; (3) primary and curative hepatectomy with a resection margin > 1 cm; (4) absence of metastasis and residual disease; (5) without preoperative adjuvant therapy; and (6) having complete follow-up information.

The clinicopathologic information including patient age, gender, alfa-fetoprotein (AFP) level, alanine aminotransferase (ALT) level, tumor size, tumor capsule, histological differentiation, liver cirrhosis, vascular invasion and necrosis were collected, which are shown in Table 1. The criteria proposed by WHO classification of Tumors of the Digestive System (2010 version) was utilized to define tumor differentiation. The American Joint Committee on Cancer/International Union Against Cancer TNM (tumor-node-metastasis) classification system (2010 version) was used to determine tumor stage. The approval was granted by the Institute Research Medical Ethics Committee of Sun Yat-sen University Cancer Center.

**Table 1:** Correlation of tumor necrosis with patients’ clinicopathological features in primary small hepatocellular carcinomas

Characteristics	Cases	Necrosis (−)	Necrosis (+)	***P*** value^a^
Gender				0.555
Male	295	155 (52.5%)	140 (47.5%)	
Female	40	23 (57.5%)	17 (42.5%)	
Age (years)				0.694
≤ 48.0^b^	166	90 (54.2%)	76 (45.8%)	
> 48.0	169	88 (52.1%)	81 (47.9%)	
AFP (ng/ml)				0.485
≤ 20	139	77 (55.4%)	62 (44.6%)	
> 20	196	101 (51.5%)	95 (48.5%)	
ALT (μ/l)				0.504
≤ 40	192	99 (51.6%)	93 (48.4%)	
> 40	143	79 (55.2%)	64 (44.8%)	
Tumor size (cm)				0.026
≤ 2.5^c^	188	110 (58.5%)	78 (41.5%)	
> 2.5	147	68 (46.3%)	79 (53.7%)	
Differentiation				0.675
Well	56	32 (57.1%)	24 (42.9%)	
Moderate	208	111 (53.4%)	97 (46.6%)	
Poor-undifferentiated	71	35 (49.3%)	36 (50.7%)	
Vascular invasion				0.003
Absent	255	147 (57.6%)	108 (42.4%)	
Present	80	31 (38.8%)	49 (61.3%)	
Envelope				0.872
Absent	214	113 (52.8%)	101 (47.2%)	
Present	121	65 (53.7%)	56 (46.3%)	
Liver cirrhosis				0. 166
Absent	201	113 (56.2%)	88 (43.8%)	
Present	134	65 (48.5%)	69 (51.5%)	

^a^Chi-square test; ^b^Median age; ^c^Median size; AFP indicates alpha-fetoprotein; ALT indicates alanine aminotranferease

### Pathological evaluation

Original histopathologic slides of the patients were previously reviewed by a senior pathologist (P Li), then independently re-confirmed by an experienced pathologist (M-Y Cai), both pathologists were blinded to the clinicopathologic prognostic data and re-examined simultaneously the slides to solve the discrepancies with a double-headed microscope. All of the selected patients had at least 3 tissue blocks, with a mean of 4.2 (median 4, range 2–8) paraffin-embedded tissue blocks per tumor available for evaluation.

The presence of tumor necrosis was carefully identified on hematoxylin-eosin (H&E)-stained slides, that characterized by homogenous clusters of sheets of dead cells, or coalescing groups of cells forming a coagulum, containing nuclear and cytoplasmic debris as previously described [19]. Coagulative tumor necrosis was found to be present without regard to the area of tumor involved, and the extent of involvement was not assessed. Vascular invasion in each HCC specimen was assessed in several serial cross sections and defined as vessel walls infiltration or the tumor emboli existence [20]. The criteria includes macroscopic and/or microscopic tumor emboli within the large capsular vessels, the central hepatic vein, or the portal vein [21].

### Follow-up

Patients were followed every 3 months by serum AFP level and ultrasound or computed tomography or magnetic resonance imaging at least every 6 months for more than 2 years after partial hepatectomy. All the enrolled patients were followed-up until January 18th, 2014. Re-resection when possible or transcatheter arterial chemoembolization, percutaneous ethanol injection or radiofrequency ablation was conducted in patients who had tumor recurrence. The definition of cancer-specific overall survival (OS) was the number of months from the date of surgery to the date of the last follow-up visit or time of death owing to sHCC. The definition of recurrence-free survival (RFS) was the number of months from the date of surgery to the first confirmability of cancer recurrence.

### Statistical analysis

Statistical analysis was computed using SPSS 19.0 software package (Chicago, IL, USA). The χ2-test was used to evaluate the correlation between tumor necrosis and the clinicopathologic parameters of the sHCC patients. For univariate analysis, Survival curves were obtained by the Kaplan-Meier method, and the differences between groups in survival were performed by the Log-rank test. Multivariate survival analyses were conducted with the Cox proportional hazard regression model. A difference of *P* <  0.05 from a two-tailed test was considered statistically significant.

## Results

### Patient characteristics

Our current study identified 335 adult sHCC patients with long-term carriers of HBV and curative surgical resection. Some patients were followed by second-line treatments at the time of recurrence. Clinicopathological characteristics of the patients are detailed in Table 1.

Of the all patients, there were 295 (88.1%) males and 40 (11.9%) females, with a median age of 48 years. 196 (58.5%) patients had serum AFP level >  20 ng/ml among the cases with pre-operation serum AFP level record. 143 (42.7%) patients had serum ALT level >  40 μ/l. The median size of the tumors was 2.5 cm. Well-differentiated or moderate-differentiated tumors were identified in a total of 264 (78.8%) patients. 214 (63.9%) tumors were encapsulated. Vascular invasion was observed in 80 (23.9%) patients. Liver cirrhosis was presented in 134 (40%) patients.

### The patterns of tumor necrosis in solitary sHCC

**Fig. 1:**
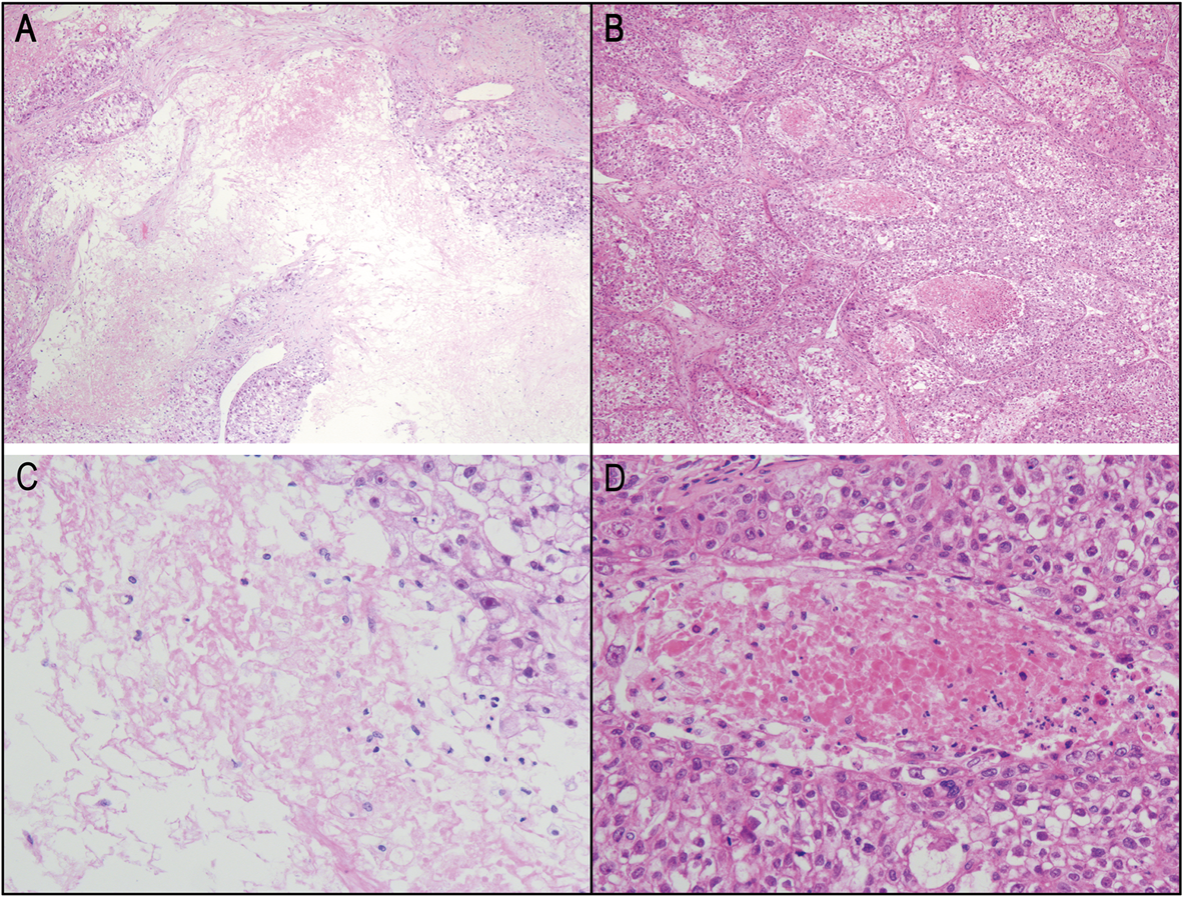
Histopathological features of tumor necrosis in primary solitary small hepatocellular carcinoma. Tumor necrosis in sHCC consisted of homogenous clusters of sheets of degenerating and dead cells, or coalescing groups of cells forming a coagulum, containing nuclear and cytoplasmic debris, with membrane integrity, intracellular organelle swelling (**a**-**b**, hematoxylin and eosin [H&E], magnification × 40; C-D, H&E, × 200)

Presence of tumor necrosis was identified in 157 of 335 (46.9%) sHCC cases (Fig. 1). Further correlation analysis demonstrated that tumor necrosis was significantly correlated with tumor size and vascular invasion in sHCC (*P* = 0.026, 0.003, respectively; Table 1).

### The relationship between tumor necrosis and patients’ survival: univariate analysis

**Fig. 2:**
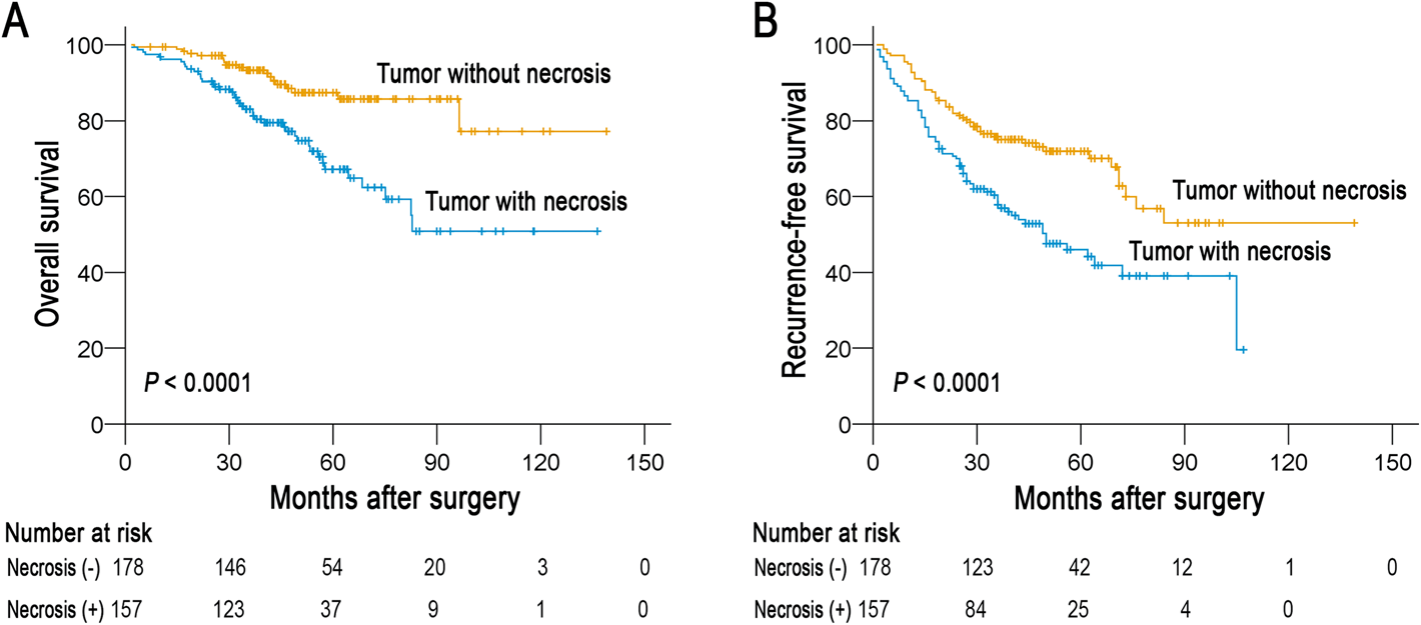
Tumor necrosis affecting postoperative survival of patients with small hepatocellular carcinoma (sHCC) (log-rank test). **a** Tumor necrosis was associated with a decrease in overall survival (OS) of patients (*P* < 0.0001). **b** Tumor necrosis was associated with a decrease in recurrence-free survival (RFS) of patients (*P* < 0.0001)

Some clinicopathological parameters indicated a significant impact of prognosis, such as tumor size (*P* = 0.001) and vascular invasion (*P* <  0.001, Table 2) in the assessment of survival of sHCC patients, as reported in our previous study [22]. The result demonstrated that patients with tumor necrosis displayed a poorer OS (Table 2; Fig. 2a) and RFS (Fig. 2b) than patients without tumor necrosis (*P* <  0.0001).

**Table 2:** Univariate and multivariate analyses of tumor necrosis and clinicopathologic variables in patients with primary small hepatocellular carcinoma^a^

Characteristics	***P*** value	Hazard Ratio (95% CI)
**Univariate analysis**		
Gender (Male vs. Female)	0.632	0.825 (0.374–1.816)
Age (≤ 48.0^b^ vs. > 48.0)	0.957	1.014 (0.615–1.672)
AFP (≤ 20 ng/ml vs. > 20 ng/ml)	0.432	1.230 (0.734–2.059)
ALT (≤ 40 μ/l vs. > 40 μ/l)	0.253	1.337 (0.812–2.201)
Tumor size (≤ 2.5^c^ cm vs. > 2.5 cm)	0.001	2.431 (1.443–4.093)
Differentiation (well-moderate vs. poor-undifferentiated)	0.512	1.215 (0.679–2.175)
Vascular invasion (absent vs. present)	< 0.001	3.033 (1.827–5.035)
Envelope (absent vs. present)	0.758	0.920 (0.544–1.559)
Liver cirrhosis (absent vs. present)	0.102	1.516 (0.921–2.495)
Tumor necrosis (absent vs. present)	< 0.001	2.821 (1.643–4.842)
**Multivariate analysis**		
Tumor size (≤ 2.5 cm vs. > 2.5 cm)	0.006	2.083 (1.229–3.529)
Vascular invasion (absent vs. present)	< 0.001	2.663 (1.598–4.437)
Tumor necrosis (absent vs. present)	0.005	2.208 (1.272–3.833)

^a^The analyses were performed with the use of Cox proportional-hazards regression; ^b^Median age; ^c^Median size; AFP indicates alpha-fetoprotein; ALT indicates alanine aminotranferease

### Multivariate Cox regression analysis

As variables examined to have prognostic influence by univariate analysis may covariate, the presence of tumor necrosis as well as other clinicopathologic features (tumor size and vascular invasion) was tested in multivariate analysis (Table 2). The presence of tumor necrosis was associated closely with poorer cancer-specific OS and RFS as evidenced by univariate (*P* <  0.001; hazard ratio, 2.821; 95% CI, 1.643–4.842) and multivariate analysis (*P* = 0.005; hazard ratio, 2.208; 95% CI, 1.272–3.833). As we described previously [22], of the other parameters, vascular invasion was evaluated as an independent prognostic factor for patient survival (*P* < 0.001; hazard ratio, 2.663; 95% CI, 1.598–4.437) and tumor size was evaluated as an independent prognostic factor for patient survival (*P* = 0.006; hazard ratio, 2.083; 95% CI, 1.229–3.529).

### The relationship between tumor necrosis and postoperative survival of sHCC patients stratified according to different risk factors

**Fig. 3:**
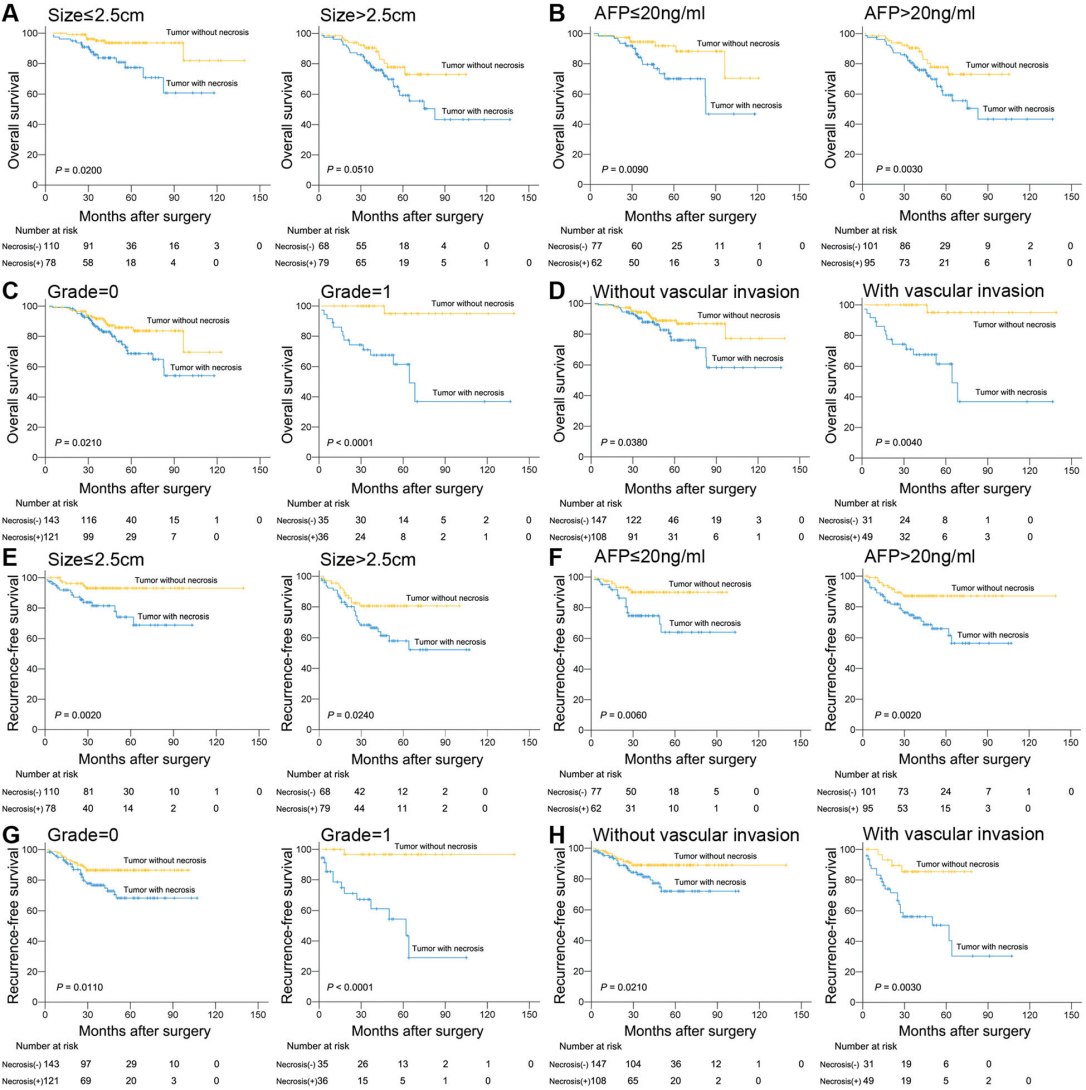
Kaplan-Meier survival curve comparing tumor necrosis affecting postoperative survival of patients with small hepatocellular carcinoma (sHCC) stratified according to different tumor size, differentiation, serum AFP level and vascular invasion. **a**, **e** Tumor necrosis was associated with a decrease in overall survival (OS) of patients with tumor size ≤2.5 cm, and a decrease in recurrence-free survival (RFS) of patients with tumor size ≤2.5 cm as well as > 2.5 cm (*P* = 0.0200, 0.0020, 0.0240, respectively). **b**, **f** Tumor necrosis was associated with a decrease in OS and RFS of patients with AFP level ≤ 20 ng/ml and >  20 ng/ml (*P* = 0.0090, 0.0030, 0.0060, 0.0020, respectively). **c**, **g** Tumor necrosis was associated with a decrease in OS and RFS of patients with different tumor differentiation (*P* = 0.0210, < 0.0001, = 0.0110, < 0.0001, respectively). **d**, **h** Tumor necrosis was associated with a decrease in OS and RFS of patients with or without vascular invasion (*P* = 0.0380, 0.0040, 0.0210, 0.0030, respectively)

Kaplan-Meier survival curve comparing tumor necrosis affecting postoperative survival of sHCC patients was stratified according to different tumor size, differentiation, serum AFP level and vascular invasion. As shown in Fig. 3a and e, tumor necrosis was associated with a decrease in OS of patients with tumor size ≤2.5 cm, and a decrease in RFS of patients with tumor size ≤2.5 cm as well as > 2.5 cm (*P* = 0.0200, 0.0020 and 0.0240, respectively). Meanwhile, tumor necrosis was associated with a decrease in OS and RFS of patients with AFP level ≤ 20 ng/ml and >  20 ng/ml (*P* = 0.0090, 0.0030, 0.0060 and 0.0020, respectively. Figure 3b and f). Tumor necrosis was associated with a decrease in OS and RFS of patients with different tumor differentiation (*P* = 0.0210, < 0.0001, = 0.0110 and < 0.0001, respectively. Figure 3c and g). Tumor necrosis was associated with a decrease in OS and RFS of patients with or without vascular invasion (*P* = 0.0380, 0.0040, 0.0210 and 0.0030, respectively. Figure 3d and h).

### New prognostic model with tumor necrosis, tumor size and vascular invasion in sHCC

**Fig. 4:**
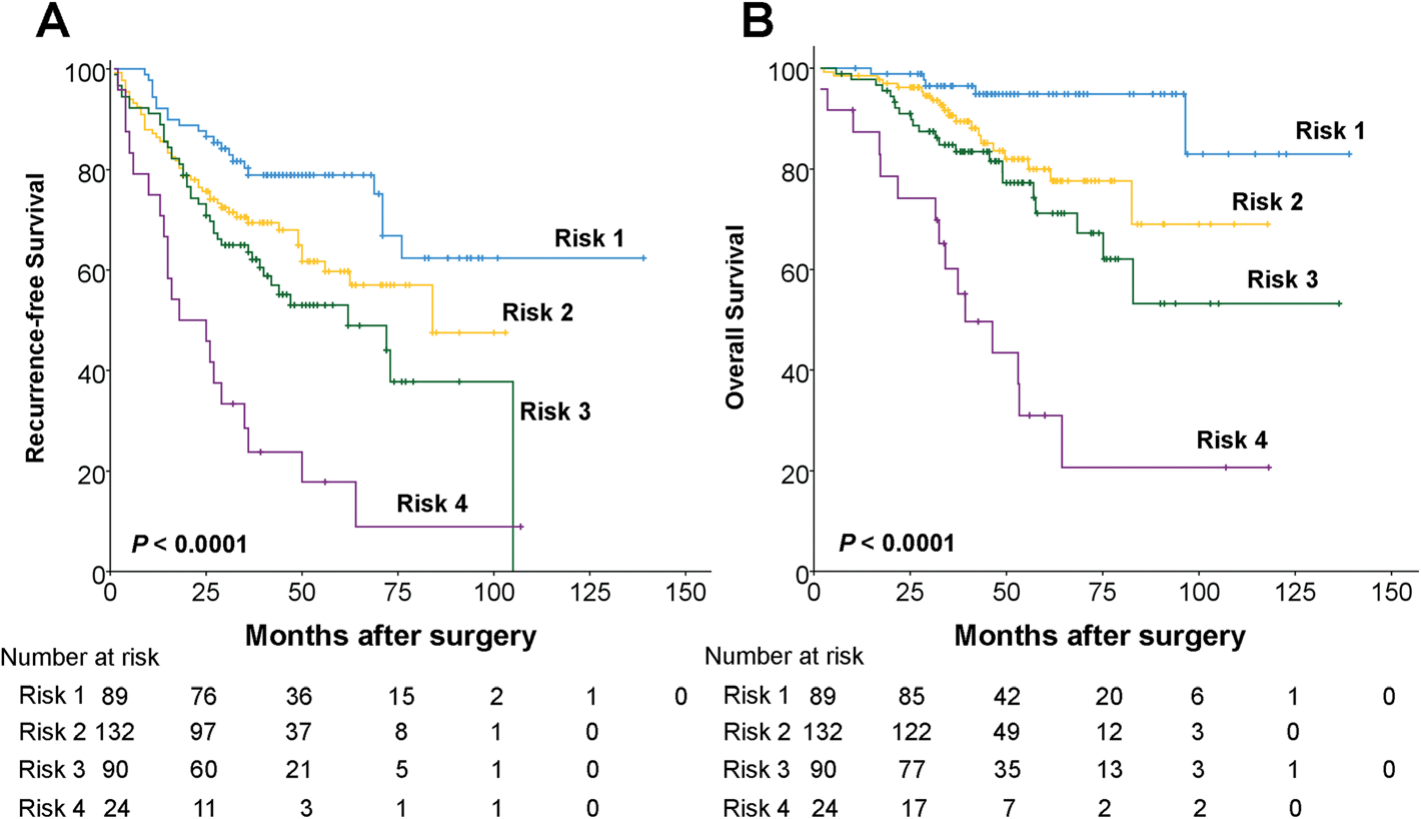
The proposed prognostic model successfully stratified risk for survival prediction of patients with sHCC (log-rank test). Using this model, these sHCC patients were stratified into four groups: risk 1, *n* = 89; risk 2, *n* = 132; risk 3, *n* = 90; risk 4, *n* = 24. **a** The RFS curves of the three groups were significantly different (*P* < 0.0001). **b** The OS curves of the three groups were significantly different (*P* < 0.0001)

According to the results of the univariate and multivariate analyses, we conducted a new clinicopathologic prognostic model combining with three poor prognostic factors: tumor necrosis, tumor size and vascular invasion. Therefore, four subtypes were designated based on the presence of the three factors (including tumor necrosis, tumor size > 2.5 cm and vascular invasion): subtype 1, absence of any risk factor; subtype 2, absence of any two risk factors; subtype 3, absence of any one risk factor; subtype 4, presence of three risk factors. This combined model could significantly stratify risk (low, intermediate and high) for OS (Fig. 4, *P* < 0.0001) and RFS (Fig. 4, *P* < 0.0001) in the current study based upon the combination of tumor necrosis, tumor size and vascular invasion.

## Discussion

In the current study, we assessed a retrospective collection of data and determined the prognostic value of tumor necrosis for pathologically proven sHCC patients. Tumor necrosis was frequently observed and a prognostic factor independent of certain well-established clinical factors, including tumor size, vascular invasion, serum AFP level and clinical stage in sHCC. Notably, we found that tumor necrosis, vascular invasion and tumor size were poor prognostic factors. Further analysis evaluated that tumor necrosis was significantly associated with vascular invasion in sHCC.

Tumor necrosis has been shown prognostic impact in lung, breast, thyroid, colorectal, pancreatic, and kidney malignancies, but also in mesenchymal tumors, such as malignant mesothelioma, gastrointestinal stromal tumors and Ewing sarcoma [13, 14, 16, 23–26]. Moreover, Soini et al. reported that the survival of sHCC patients showing a high proliferation and simultaneously a low degree of apoptosis and necrosis was significantly shorter [17]. Martino et al. found that the non-cirrhotic HCC patients may presented with certain areas of necrosis but did not demonstrate the relative prognosis [18]. Thus, data regarding the incidence and prognostic impact of necrosis in sHCC remain limited. Our research has characterized and demonstrated for the first time that tumor necrosis predicts poor survival in sHCC, which implies a relationship that increased tumor cell death indicates a more aggressive cancer. Tumor microvessels are fragile and susceptible to hypoxia, suggesting that the degree of tumor necrosis reflects the level of intratumoral hypoxia [13, 14, 27, 28]. Intratumoral hypoxia has been reported to correlate with poor prognosis and sensitive to radiotherapy and chemotherapy in solid tumors with a polarographic needles [13]. In breast cancer, tumor necrosis has been shown to correlate with increased tumor size, high-grade disease, high microvessel density, and infiltrates of macrophages that express vascular endothelial growth factor, suggesting that hypoxic environment causing tumor necrosis stimulates angiogenesis owing to angiogenic growth factors released by infiltrating macrophages in rapidly growing tumors [23, 29, 30].

Vascular invasion is found to be related to decreased overall and recurrence free survival in post-surgical resection and transplant HCC patients [31, 32]. Our previous study revealed evidence supporting that vascular invasion had an adverse impact on long-term survival in sHCC patients [22]. The association of tumor necrosis and vascular invasion in the current study is consistent with studies in breast cancer and malignant mesothelioma, in which it was also observed that microvessel hot spots were situated away from areas of tumor necrosis [23, 33]. It can be reasonably explained this paradoxical relationship that because of tumor rapid growth the microvasculature is ischemic damaged due to its supply overload and thereby increasing tumor necrosis. Tumor size is a well-known risk factor for poor survival of solid tumor. In breast cancer, tumor necrosis correlated with increasing tumor size [23]. It was also confirmed that increasing mass was associated with hypoxia in the experimental murine allograft model [34]. Similarly, we previously demonstrated that tumor size > 2.5 cm was correlated with a worse OS or RFS and tumor necrosis was associated with increasing tumor size in the sHCC patients [22].

According to the clinical practice guideline, surgical resection is recommended in early stage HCC [35]. However, sHCC patients still suffer from tumor recurrence due to various intratumour heterogeneity of the patient population. Traditionally, the pTNM Staging and grading system have been utilized to determine the treatment and the prognosis of solid tumors. Nevertheless, based on specific clinicopathologic features and extent of disease, this system might have their limitation for sorting out high risk of tumor recurrence populations from early-stage HCC patients and offering clear pathways to help novel follow-up strategies and salvage therapy. Thus, there is a need for new objective strategies that can effectively distinguish between sHCC patients with favorable and unfavorable outcome. By far, in renal cell carcinomas, the presence of tumor necrosis has been proposed to be incorporate into grade classification system [36]. In our present study, when OS and RFS rates were compared between patients with and without tumor necrosis, significantly poorer prognosis could be observed for those with tumor necrosis in one large population-based cohort of sHCC patients, supporting the concept that tumor necrosis adds additional prognostic information and could further stratify patients with or without aggressive clinical course and/or adverse outcome. In addition, we proposed a combined prognostic model with tumor necrosis, tumor size and vascular invasion and found that the model may be a useful prognostic index to reflect the aggressive phenotype for sHCC. Generally, our findings support the idea that the pTMN supplemented by tumor necrosis, vascular invasion and tumor size might improve the ability to discriminate sHCC patients’ outcome, along with the effectiveness of personalized and precise treatment.

The retrospective nature of this study may be considered its major limitation. There are also strong efforts to integrate biomarkers into established clinicopathologic models to further improve their predictive ability. However, our study was strengthened by the fact that all of the histopathological slides were re-evaluated by two gastrointestinal pathologists. Although, we believe that our results contribute to the literature because it includes only patients with sHCC.

## Conclusions

In summary, our findings demonstrated that tumor necrosis could be used as an additional effective predictor in identifying those patients at increased risk of tumor progression. The proposed new prognostic model (combined tumor size, vascular invasion and tumor necrosis) might improve the ability to discriminate sHCC patients’ outcome, along with the effectiveness of personalized and precise treatment in the future.

## Data Availability

The data that support the findings of this study are available from Sun Yat-sen University Cancer Center, but restrictions apply to the availability of these data, which were used under license for the current study, and so are not publicly available.

## Abbreviations

sHCC: Small Hepatocellular Carcinoma
HBV: Hepatitis B Virus
AFP: Alfa-fetoprotein
ALT: Alanine aminotransferase
OS: Overall survival
RFS: Recurrence-free survival
